# miR205 inhibits stem cell renewal in SUM159PT breast cancer cells

**DOI:** 10.1371/journal.pone.0188637

**Published:** 2017-11-28

**Authors:** Víctor Mayoral-Varo, Annarica Calcabrini, María Pilar Sánchez-Bailón, Jorge Martín-Pérez

**Affiliations:** Department of Cancer Biology, Instituto de Investigaciones Biomédicas A. Sols (CSIC/UAM), 4 Arturo Duperier, Madrid, Spain; University of South Alabama Mitchell Cancer Institute, UNITED STATES

## Abstract

miR205 has a dual activity, as tumor suppressor and as oncogene. Here we analyzed the impact of miR205 ectopic expression in the initial tumorigenic processes of SUM159PT, a triple negative breast cancer cell line with low endogenous levels of miR205. In SUM159PT, miR205 inhibited expression of its targets VEGFA, ErbB3, Zeb1, Fyn and Lyn A/B; it reduced cell proliferation, and Myc/cyclin D1 levels, while increased p27^kip1^ expression. miR205 abolished anchorage-independent growth, inhibited migration and invasion, Src-kinases/Stat3 axis activation, and levels of secreted MMP9. miR205 also reduced expression of CD44 and TAZ, E2A.E12, Twist, Snail1 and CK5, associated with epithelial-mesenchymal transition (EMT). Importantly, we show that miR205 inhibited SUM159PT cancer-stem cell renewal, expression in mammospheres of CD44 and ALDH1 stem-cell markers, TAZ, and E2A.E12. All these effects of miR205 were reverted by Anti-miR205 co-expression, demonstrating its specificity. Thus, all these results strongly suggest that ectopic expression of miR205 in SUM159PT affected several parameters associated with initial steps of tumorigenesis.

## Introduction

MicroRNAs (miRs) are small noncoding RNAs that usually hybridize to 3’ UTR of mRNAs facilitating their degradation, resulting in reduced expression of the encoded proteins [1]. miRs control many cellular functions in eukaryotic organisms, including development, differentiation, proliferation, apoptosis, etc. [2]. Deregulation of miRs expression has been associated with cancer, including breast tumors [3]. microRNA signature is associated with breast cancer metastases, where miR450a, miR148a, miR30b, miR150, and miR155 are overexpressed and miR99b, miR125b, miR205, miR130b, miR24 and miR99a are down-regulated [4]. In triple-negative breast cancer (TNBC), tumor that does not express receptors for estrogens, progesterone, and does not overexpress Her2 (ER^-^, PR^-^, Her2^-^)[5], expression of miR10b, miR122, miR145, and miR205 is lower than in normal tissue, suggesting that they act as tumor-suppressors [6].

miR205 is expressed in the myoepithelial/basal cell compartment of mammary ducts and lobules, and it is highly reduced in the basal tumors and in TNBC cell lines [7]. Experimental data support the dual actions of miR205 both as a tumor suppressor by targeting ErbB3, VEGFA, ZEB1/2, etc., in breast, melanoma, renal, glioblastoma and lung cancer, and as a tumor promoter by regulating PTEN, TRAF2 and SHIP2 in breast cancer, nasopharyngeal carcinoma, and lung squamous cell carcinoma [8]. miR205 inhibits epithelial-mesenchymal transition (EMT), by targeting ZEB1/2 [9], and suppresses tumor expansion from basal membrane to stroma [6].

Here we analyzed the effects of miR205 ectopic expression on initial steps of breast tumorigenesis and metastasis using SUM159PT (SUM159 from now on). SUM159 cells were derived from a primary human anaplastic breast carcinoma, they are ER^-^, PR^-^, Her2^-^ (TNBC), and not only has a mutated p53, as MDA-MB-231 cells, but also PIK3CA [10, 11]. SUM159 cells exhibit a spindle-like appearance, consistent with basal-B/claudin-low classification of TNBC, and can also readily form mammospheres in culture and metastasize *in vivo* [5, 10, 12–16]. Thus, they are considered as a good model of TNBC cells. We observed that miR205 inhibited cell proliferation, migration, invasion, anchorage-independent growth, and more importantly, tumor-initiating/cancer-stem cells self-renewal. All these effects were reversed by Anti-miR205 co-expression, supporting the specificity of miR205. Together these results suggest that miR205 could affect SUM159 tumorigenicity by inhibiting cancer stem cell renewal.

## Materials and methods

### Reagents

Antibodies to c-Myc (sc-7274), cyclin D1 (sc-753), ErbB-3 (sc-285), Lyn A/B (sc-764), Fyn (sc-16), Src2 (sc-18), VEGF-A (sc-53462), E2A.E12 (sc-349), and ZEB1(sc-10572) (Santa Cruz Biotechnology), p27^Kip1^ (BD-Pharmingen 554069), ALDH1 (BD, 661194), Stat3 (BD-Transduction Laboratories, S21320), Twist1/2 (Gene Tex, GTX127310), pY705-Stat3 (Cell Signaling Technology, #9131), Snail-1 (Cell Signaling Technology, LF062), CK5 (ABCAM, ab52635), pY418-Src (Invitrogen, 44660G), GAPDH (MAB374) and MMP9 (AB19016) (Millipore), PARP (Biomol, SA-249 clone C-2_10), β-actin (A5441), TAZ (HPA007415), and hydrocortisone were from Sigma-Aldrich, and CD44 (clone HP 2/9) was a gitf from Dr. F. Sanchez-Madrid (University Hospital La Princesa, UAM) [17], Matrigel^TM^ (Corning). Secondary horseradish peroxidase-conjugated antibodies, and B27 (Life Technologies). EGF, and bFGF (PeproTech EC Ltd). Fetal Calf Serum (FCS), Acrylamide/Bisacrylamide, SDS and ammonium persulfate (Bio-Rad Laboratories). ECL (GE Healthcare Biosciences). BCA protein assay (Thermo Scientific).

### Cell lines and culture

SUM159PT were provided by Dr. G. Dontu (King's College London School of Medicine, UK) [18]. SUM159 cells were mycoplasma free, and they were authenticated by means of short-tandem repeat (STR) analysis (GenePrint 10 System and Gene Mapper software, Applied Biosystems, and PowerPlexW 1.2 System, Promega, respectively). Profiles obtained were checked against public data bases ATCC and DSMZ (German Collection of Microorganisms and Cell Cultures). SUM159 were cultured in Ham’s F12, 5% FCS, 5μg/ml insulin, 1μg/ml hydrocortisone, 2mM glutamine, 100IU/ml penicillin, and 100μg/ml streptomycin.

### Generation of SUM159 expressing miR205 and Anti-miR205

SUM159 were transfected either with pEP-empty vector (control vector) or with pEP-miR205 (Cell Biolabs, Inc.) by calcium phosphate precipitation, and selected 48h later with 2μg/ml puromycin. Resistant clones were tested for miR205 expression by qRT-PCR, and the pool of positive clones was used for experiments. Pools of SUM159_Control (Ctrl) and SUM159_miR205 were then infected with the bicistronic lentivirus mir205Zip (miR205-IRES-EGFP, Gentaur) to generate SUM159_miR205_Anti-miR205 and SUM159_Control_Anti-miR205 cells. Subsequently, EGFP-positive cells were isolated by *Fluorescence-activated cell sorting* (FACS-Vantage SE, BD) to set the SUM159_miR205_Anti-miR205 cell line. The SUM159_Control_Anti-miR205 cells were collected as a pool after lentiviral transduction. Cells were maintained in Ham’s F12, 5% FCS, insulin (5μg/ml), hydrocortisone (1μg/ml), glutamine (2mM), penicillin (100IU/ml), streptomycin (100μg/ml) and puromycin (0.2μg/ml).

### RNA preparation, qRT-PCR of miR205

RNA was isolated from three independent experiments performed in triplicate using RNeasy kit (Qiagen). After testing for RNA integrity, triplicate RNAs from each experiment were pooled. miR205 expression was determined by qRT-PCR using TaqMan^TM^ probe for hsa-miR205 (#4427975), and TBP (Tata Box Binding Protein, Hs00427621_m1) as endogenous control (Life Technologies). Relative miR205 expression was calculated as the difference in cycle threshold (ΔCt) between target gene and TBP respectively; ΔΔCt was the difference between ΔCt values of test sample and that of control. Relative expression of target genes was calculated as 2^-ΔΔCt^.

### Cell proliferation

Cell proliferation was evaluated by counting viable cells performing Trypan blue (Sigma-Aldrich) exclusion assay. SUM159_Control (Ctrl), SUM159_miR205 (miR), SUM159_Control-Anti-miR205 (Ctrl_Anti-miR), and SUM159_mir205_Anti-miR205 (miR_Anti-miR) cells were seeded at 3x10^5^ cells/60mm dishes, 72h later they were trypsinized, cells were pelleted and resuspended in culture medium, mixed with a 0.4% Trypan blue/PBS solution (1:1), loaded on a hemocytometer, and Trypan blue-negative (viable cells) and Trypan blue-positive cells (dead cells) were counted.

### Anchorage-independent growth

Cells were resuspended in warmed solution of 0.3% agarose in complete medium and seeded at 10^5^cells/60mm dishes with a bottom layer of 0.5% agarose. Cells were re-fed every 72h with complete medium (300μl/dish). At 10-day growth, plates were stained with 0.5 ml of 0.005% crystal violet/water for 1h and colonies with diameter ≥ 0.1 mm from 4–5 fields/plate were counted.

### Immunoblotting

Cell lysis and immunoblotting analyses were performed as previously described [19].

### Cell migration

Cells were seeded in complete medium in 6-well plate and grown to confluence. The monolayer was scratched with a 200μl micropipette tip, and washed with fresh medium to remove floating cells. Complete medium was added to cultures, and photomicrographs were taken every 30min with a Microscope Cell Observer Z1 system (Carl Zeiss AG) equipped with controlled environment chamber and Camera Cascade 1k to monitor wound closure. Migration was quantified using wound-healing tool of ImageJ, as described [20].

### Invasion assay

Invasiveness was determined as described [20]. Briefly, cells were seeded in serum-free medium on the upper chamber of cell culture inserts of 24-well plate (8μm-pore PET membranes, BD) coated with Matrigel^TM^ (5x10^4^/well/200μl). The lower chamber was filled with 600μl of 20% FBS; 22h later, after removing cells on top of inserts, those on lower surface were fixed, nuclei stained with DAPI and mounted on slides with Prolong antifade-reagent. Filters were observed with a Plan 20x/0.50 objective of axiophot fluorescence microscope (Zeiss, Germany) equipped with Olympus DP70 digital camera. DAPI-stained nuclei were counted.

### Mammosphere cultures

Single cell suspensions of adherent cultures were plated in 6-well ultralow attachment plates (Falcon, Corning Life Science) at 2x10^3^cells/well. Mammosphere cultures were maintained in serum-free DMEM/F12 media (1:1), B27 (1:50), EGF (20ng/ml) and bFGF (20ng/ml), insulin (5μg/ml), hydrocortisone (5μg/ml). After 10 days, mammospheres (sphere-like structures with diameter ≥50μm) were clearly detected by optical phase contrast microscope (Nikon-Eclipse TS100, 4x magnification). Cultures were then pipetted up and down to induce sphere dissociation in single cells, which were seeded again for mammosphere formation. The experiment ended at third generation of mammosphere formation. Sphere Forming Efficiency (SFE) was then calculated as number of spheres formed per number of seeded cells and expressed as % means ± SD.

### Statistical analyses

Mean values, standard deviation and statistical significance between data from two different experimental conditions were determined by two-tail Student *t*-test.

## Results

### Targets validation of miR205

**Fig 1:**
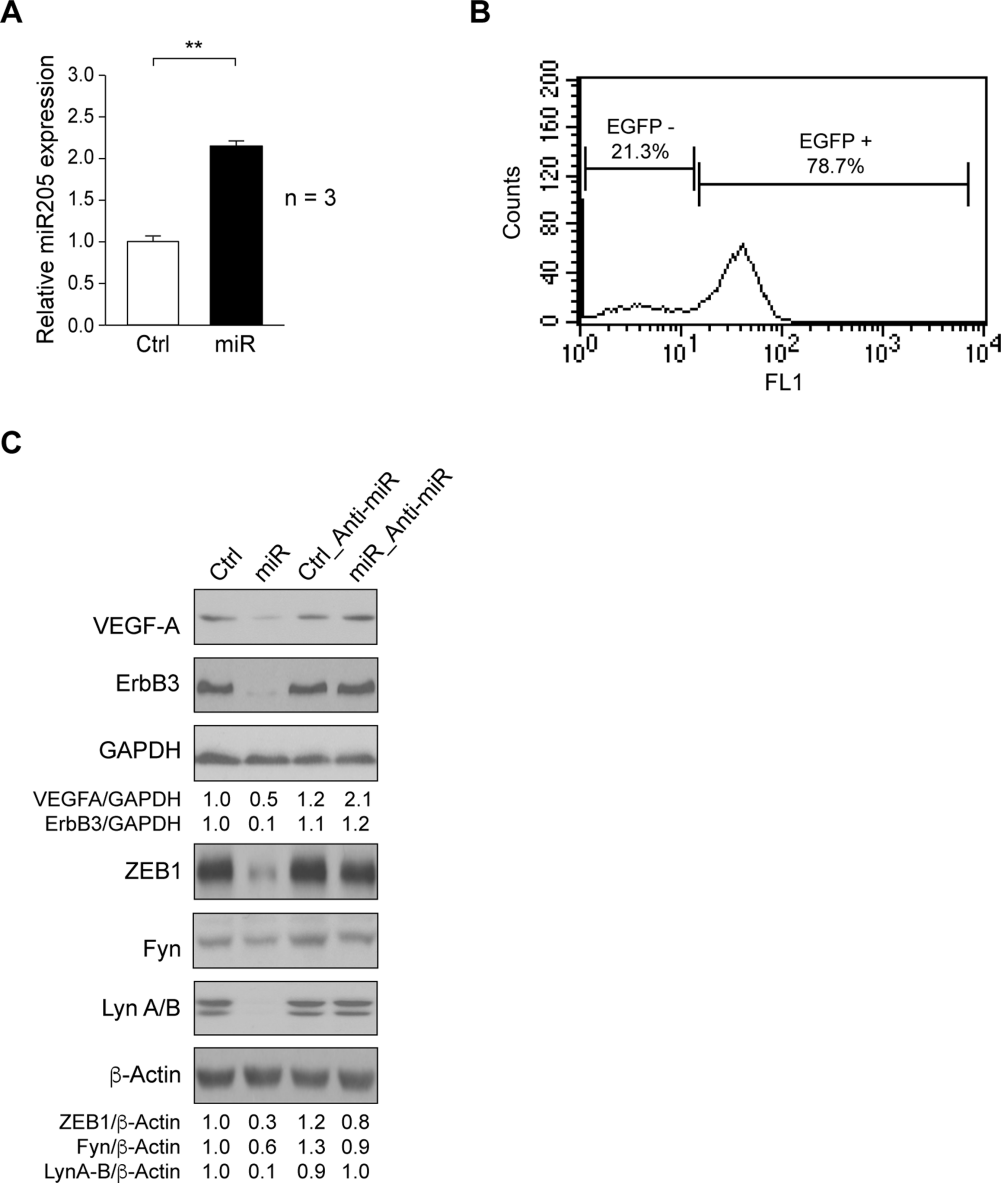
Validation of miR205 targets. (A) miR205 expression determined by a TaqMan^TM^ assay in SUM159_Control (Ctrl), and SUM159_miR205 (miR) (see Materials and Methods). Results are shown as mean ± SD of relative miR205 levels in three independent experiments in triplicate, considering arbitrarily the first sample of SUM159_Control (Ctrl) as 1 (**p<0.01). (B) EGFP-positive cells were isolated by FACS-Vantage SE from SUM159_miR205 infected with a bicistronic lentivirus containing Anti-miR205/EGFP to establish SUM159_miR205_Anti-miR205 cell line (see Materials and Methods). Result is shown as histogram from background (EGFP-) and positive populations (EGFP+) of sorted SUM159_miR205_Anti-miR205 cells. (C) Immunoblotting detection of VEGF-A, ErbB3, ZEB1, Fyn, and Lyn A/B from extracts of SUM159_Control (Ctrl), SUM159_miR205 (miR), SUM159_Control_Anti-miR205 (Ctrl_Anti-miR), and SUM159_miR205_Anti-miR205 (miR_Anti-miR) cells; GAPDH or β-actin were determined for loading controls. These are representative results from 3 independent experiments. The ratios referred to SUM159_Control (Ctrl) considered as 1.

Here we determined the effects of miR205 ectopic expression in SUM159 cell line. Cells were transfected with pEP-empty vector (Control) or pEP-miR205 by calcium phosphate precipitation and selected with puromycin (2μg/ml). Positive clones were pooled, and miR205 expression was analyzed by qRT-PCR. miR205 expression was significantly increased in SUM159_miR205 (miR) as compared to SUM159_Control cells (Ctrl) (Fig 1A). To verify the specificity of miR205 effects, SUM159_Control (Ctrl) and SUM159_miR205 (miR) were transduced with a bicistronic IRES lentivirus expressing Anti-miR205 and EGFP. It should be noted that SUM159 cells endogenously expressed basal levels of miR205 [21]. While SUM159_Control_Anti-miR205 (Ctrl-Anti-miR) cells were collected as a pool, SUM159_miR205_Anti-miR205 (miR-Anti-miR) cells were isolated by three consecutive rounds of selection by FACS on the basis of EGFP signal (Fig 1B).

Among the multiple targets of miR205 in metastatic breast cancer, VEGF-A, ErbB3, and ZEB1 have been described [9, 22]. We tested for their expression by immunoblotting in cell extracts of exponential growing SUM159_miR205 (miR) and SUM159_Control (Ctrl). Results showed that miR205 reduced VEGF-A, ErbB3, and ZEB1 levels as compared to control-cells (Fig 1C). Since in A498 renal cancer cells [23] miR205 reduced the levels of c-Src, Yes and Lyn A/B, we analyzed its effects in SUM159 cells, and found that it induced reduction of Fyn and Lyn A/B levels as compared to control (Fig 1C). All these effects were due to miR205, as expression of these targets recovered by the counteracting effect of Anti-miR205 (Fig 1C).

### Role of miR205 in cell proliferation and anchorage-independent growth

**Fig 2:**
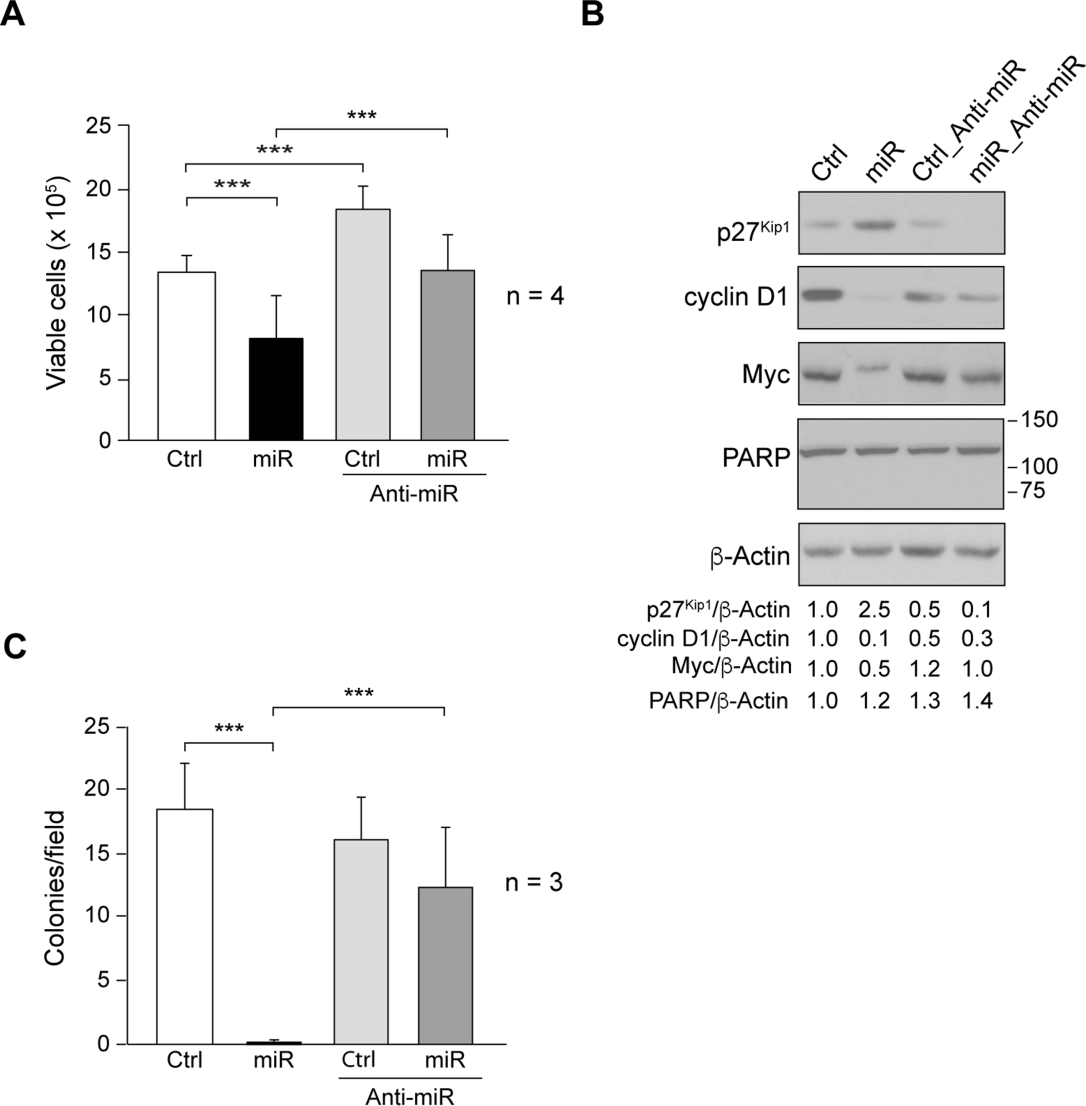
Role of miR205 in cell proliferation and anchorage-independent growth. (A) Cell proliferation was determined by performing Trypan blue exclusion assay in SUM159_Control (Ctrl), SUM159_miR205 (miR), SUM159_Control_Anti-miR205 (Ctrl_Anti-miR), SUM159_miR205_Anti-miR205 (miR_Anti-miR) cells. Cells were seeded at 3x10^5^ cells/60mm dishes, and 72h later both Trypan blue-negative (viable cells) and Trypan blue-positive cells (dead cells) were counted (see Materials and Methods). Results are shown as measurements of Trypan blue-negative cells, mean ± SD from four independent experiments in triplicate. (B) Immunoblotting detection of p27^Kip1^, cyclin D1, Myc, and PARP in cell extracts. Membranes were reblotted with anti-β-actin for loading control. Results are representative of three independent experiments. The ratios referred to SUM159_Control (Ctrl) considered as 1. (C) Number of colonies/field obtained after cell growth in soft-agar for 10 days (see Material and Methods). Average of colony number/field ± SD from three independent experiments in triplicate. (***p<0.001).

The effects of miR205 in SUM159 proliferation were evaluated by counting viable cells by performing Trypan blue exclusion assay after 72h of plating 3x10^5^ cells/60mm dishes, as described in Materials and Methods. miR205 (miR) significantly reduced cell proliferation as compared to control (Ctrl). Interestingly, since Anti-miR205 blocked basal miR205 endogenous expression previously described [21], SUM159_Control_Anti-miR205 (Ctrl_Anti-miR) increased the number of viable cells as compared to SUM159_Control (Ctrl) (Fig 2A). The percentage of Trypan blue stained cells (dead cells) was always lower than 5% (data not shown), indicating that miR205 cytotoxicity was negligible. Moreover, miR205 did not induce apoptosis; cell cycle analyses by propidium iodide did not show the presence of sub-G1 cells (data not shown), and PARP degradation was undetected (Fig 2B). Consistently, miR205 increased cellular levels of p27^Kip1^ and reduced those of Myc, and cyclin D1 (Fig 2B). These effects were antagonized by Anti-miR205 (Anti-miR) co-expression (Fig 2B).

Anchorage-independent growth was determined by colony formation in soft agar (see Materials and Methods), and it is usually correlated with cellular tumorigenic and metastatic potential, a typical feature of cancer aggressive phenotype of TNBC in vivo. In SUM159, expression of miR205 (miR) abolished SUM159 colony formation, inhibition that was counteracted by Anti-miR205 (Anti-miR) co-expression (Fig 2C), suggesting the involvement of miR205 in reducing cell tumorigenicity.

### Effect of miR205 on SUM159 migration and invasion

**Fig 3:**
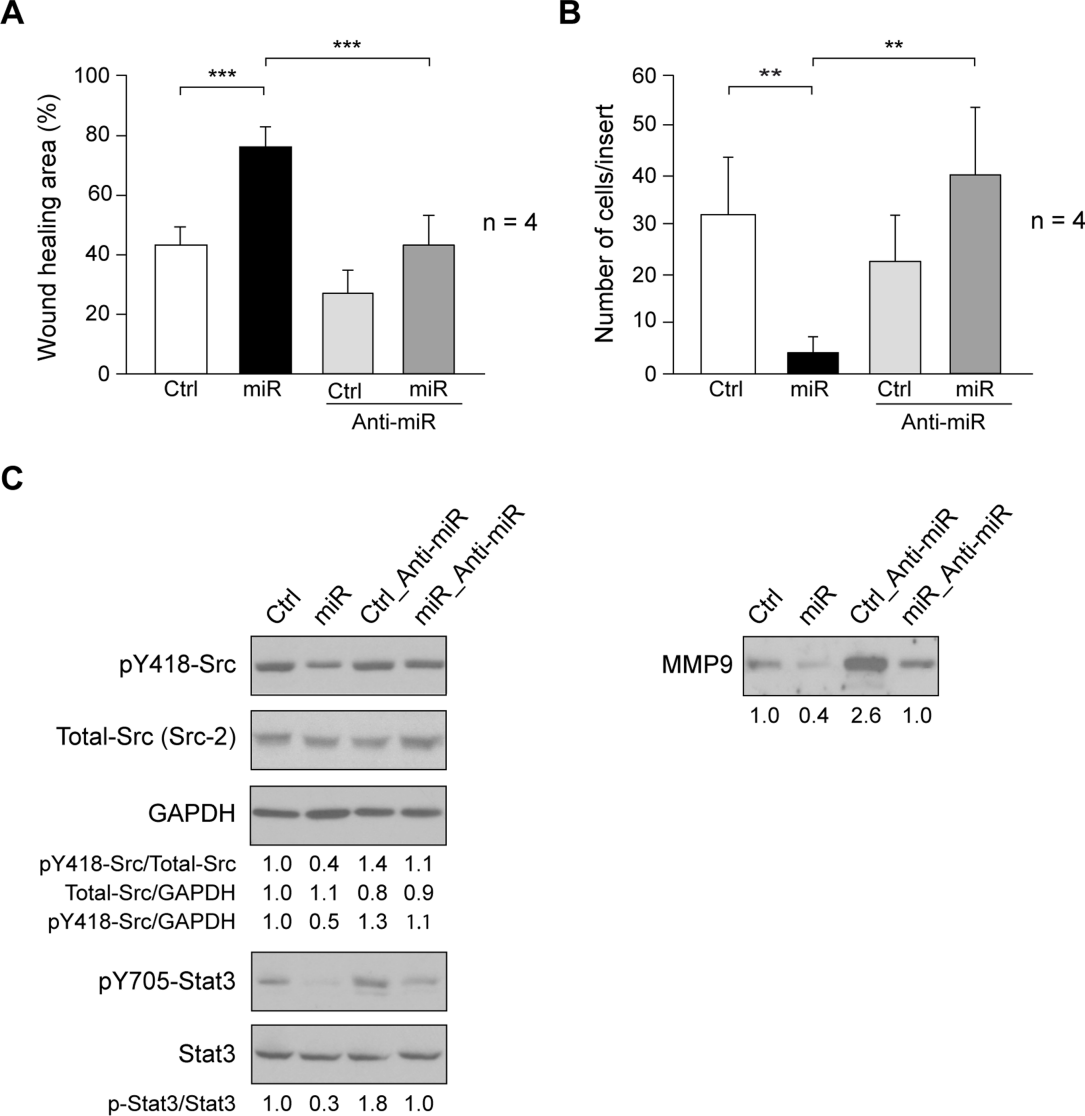
Effect of miR205 expression on cell migration and invasion. (A) Cell migration was determined by wound-healing assay in SUM159_Control (Ctrl), SUM159_miR205 (miR), SUM159_Control_Anti-miR205 (Ctrl_Anti-miR), SUM159_miR205_Anti-miR205 (miR_Anti-miR) cells (see Materials and methods). Results are expressed as mean percentage of wound healing area ± SD at 20h respect to 0h from three independent experiments (***p < 0.001). (B) Cell invasion through Matrigel^TM^-coated inserts (see Materials and methods). The number of invaded cells per insert is shown as average ± SD of four experiments in triplicate. (**p<0.01). (C) Immunoblotting detection of activated SFKs (pY418-Src), Total-Src (Src2), and GAPDH as loading control, as well as pY705-Stat3, and Stat3 in cell extracts, and of MMP9 in cellular secretome from SUM159_miR205 and Sum159_Control cells containing equal amount of total proteins. They are representative results from 3 independent experiments carried out in triplicate. The ratios referred to SUM159_Control (Ctrl) considered as 1.

Overexpression of miR205 in A498 cells reduced activation of Src/Stat3 pathway that is associated to cell migration and invasion [23]. In SUM159, ectopic expression miR205 (miR) significantly reduced migration, as compared to control-cells, inhibition that was neutralized by Anti-miR205 (Anti-miR) co-expression (Fig 3A).

Reduced levels of miR205 are associated with lymphoid node metastasis in TNBC [24]. We determined if miR205 altered SUM159 invasion in Matrigel^TM^, and found a significant inhibition of invasion, as revealed by the reduced number of invading cells when compared with control-cells (Fig 3B). Since activation of Src/Stat3 axis promotes cell invasion, we tested for activation of SFKs by determining the autophosphorylation site of Src kinases (pY418-Src), which is highly conserved among SFKs. The immunoblotting membrane was reblotted with Src2 that recognizes the ubiquitous Src family members, c-Src, Yes, and Fyn, as well as by GAPDH, a constant expression gene, as loading control. These analyses showed that autophosphorylation/activation of Src kinases was reduced in SUM159_miR205 (miR) as compared to control-cells (Ctrl) (Fig 3C). Consistently, phosphorylation of Stat3 at Y705 was also inhibited by miR205 (miR); Anti-miR205 (Anti-miR) co-expression reversed these effects (Fig 3C). Moreover, MMP9 expression is associated with invasive breast tumors [22, 25], we determined the expression of MMP9 in the secretome of SUM159_miR205 (miR) and Sum159_Control (Ctrl) cells containing equal amount of total proteins. Consistently with reduced invasiveness, MMP9 was diminished in the secretome of SUM159_miR205 (miR), as compared to SUM159_Control (Ctrl); Anti-miR205 (Anti-miR) co-expression counteracted these effects (Fig 3C). Since, SUM159_Control (Ctrl) cells have endogenous basal levels of miR205 (miR), co-expression of Anti-miR205 (Anti-miR) consistently increased levels of MMP9 in the secretome. Inhibition of Src/Stat3 axis, and reduction of MMP9 support the effect of miR205 in decreased invasiveness of SUM159.

### Role of miR205 on tumorigenic cell renewal

**Fig 4:**
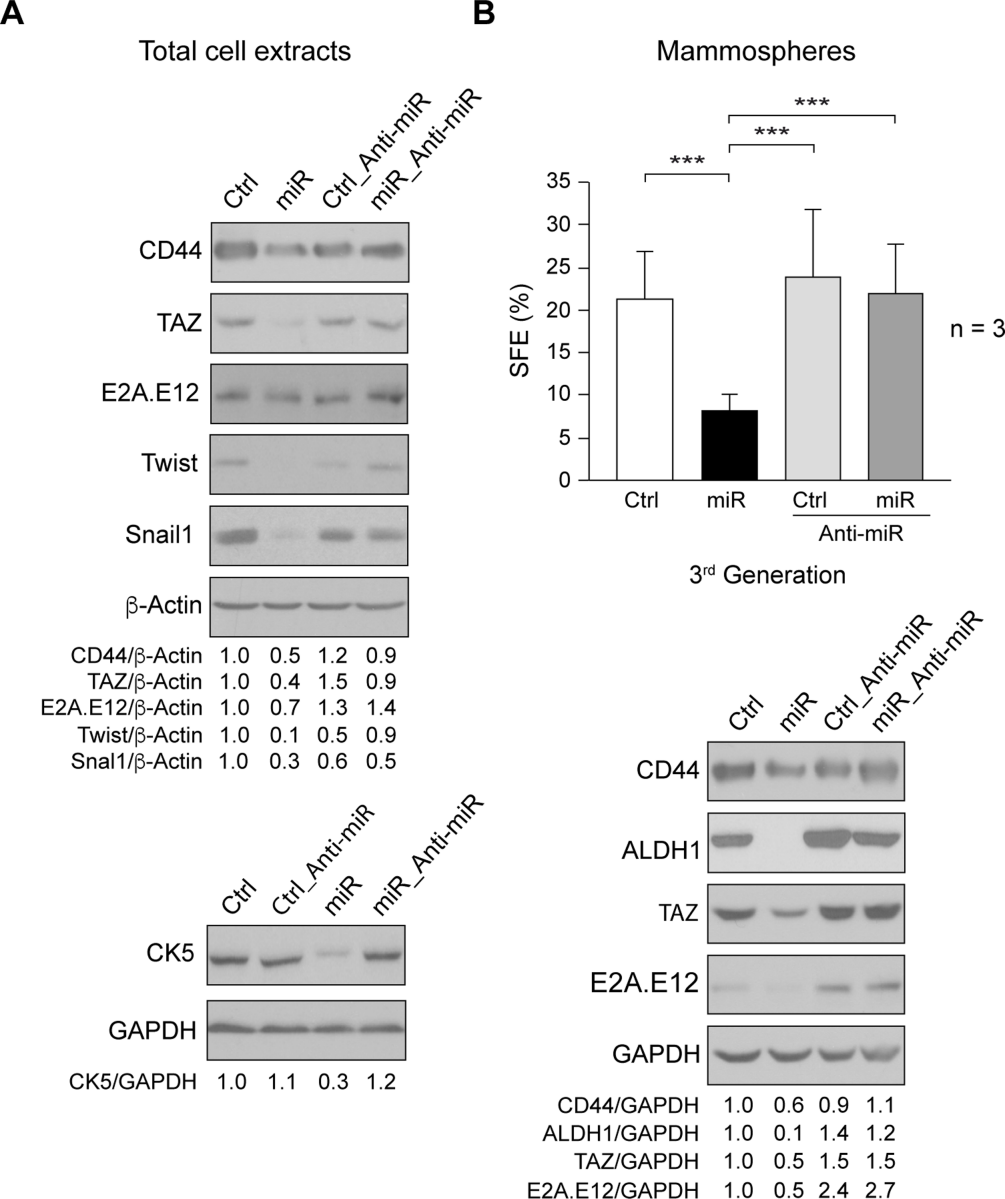
Role of miR205 in mammospheres formation. (A) Cell extracts from exponentially growing SUM159_Control (Ctrl), _miR205 (miR), _Control_Anti-miR205 (Ctrl_Anti-miR), _miR205_Anti-miR205 (miR_Anti-miR) cells were used to determine expression of CD44, TAZ, E2A.E12, Twist, Snail1, and CK5 by immunoblotting, β-Actin and GAPDH were assessed as loading control. (B) Cells plated at 2x10^3^ cells/well were cultured in conditions for mammosphere growth, after 10 days, mammospheres were disaggregated and seeded again, and at the third passage, the Spheres Formation Efficiency (SFE) was determined (see Material and Methods). The results are represented as percentage of SFE ± SD from three independent experiments in triplicate. (***p<0.001). Immunoblotting detection in mammospheres extracts of CD44, TAZ, E2A, and ALDH1, GAPDH was used as loading control. They are representative results from three independent experiments. The ratios referred to SUM159_Control (Ctrl) considered as 1.

A small subpopulation of tumorigenic cells within total tumor mass is responsible for cancer initiation and maintenance [15, 26, 27]. Our results showed that miR205 reduced proliferation, migration and invasion properties of SUM159 cells, suggesting that it could be involved in regulating cell tumorigenicity. Then, we analyzed expression of markers associated with epithelia-mesenchymal process in the whole cell population, and found that miR205 (miR) reduced expression of CD44, TAZ (WWTR1, transcriptional co-activator with PDZ-binding motif), E2A.E12, Twist, and Snail-1, as well as of the mesenchymal CK5 (cytokeratin 5) (Fig 4A). Nevertheless, while several mesenchymal markers were reduced in SUM159_miR205, we were unable to detect E-cadherin expression.

We next enriched and characterized the cancer-initiating/cancer-stem cell subpopulation by analyzing their capacity of self-renewal (spheres formation efficiency, SFE), which is determined by the ability to form mammospheres (see Materials and Methods). Expression of miR205 (miR) in SUM159 cells significantly reduced SFE in the third generation of mammospheres, as compared to control-cells (Ctrl) (Fig 4B). Co-expression of Anti-miR205 (Anti-miR) reversed SFE inhibition, indicating that miR205 caused this effect. To exclude mammospheres formation by cell aggregation, SFE was also performed at lower density (10cells/well) in 96-well plates, with similar results (data not shown). Consistently, the levels of cancer stemness and mesenchymal markers ALDH1 (Aldehyde Dehydrogenase 1), CD44, TAZ, and E2A-E12 were reduced in mammospheres by miR205 (miR), and were recovered by Anti-miR205 (Anti-miR) co-expression (Fig 4B). Thus, miR205 inhibited self-renewal of the cancer-stem cells derived from SUM159 cells. Together these data support the concept that this micro-RNA acts as an anti-oncogene in SUM159 cells.

## Discussion

miR205 is detected in the cytoplasm of myoepithelial cells in lobules and ducts of normal mammary gland tissue, while in TNBC is almost absent [6–8]. Since the TNBC tumors do not express PR, ER and do not overexpress Her2 [5], they have not the classical targets for therapeutic actions, and most of them are very aggressive and metastatic [28]. Here we analyzed the effects of ectopic expression of miR205 in the TNBC cell line SUM159 [10, 12–16]. Moreover, to confirm the specificity of its actions, SUM159_Control and SUM159_miR205 cells were transduced with a bicistronic-lentivirus containing Anti-miR205/EGFP. Among the multiple miR205 target genes [29], ErbB3 and VEGFA expression was repressed by miR205 in MDA-MB-231 cells [22]. These results are in agreement with the reduced expression of ErbB3 and VEGFA observed here after ectopic expression of miR205 in SUM159 cells. In TGF-β-treated MDCK cells and in esophageal squamous cell carcinoma cells, miR205 controls expression of ZEB transcription factors [9, 30]. Also in our cell model miR205 suppressed expression of ZEB1, as well as E2A.E12 and Twist, transcription factors involved in EMT [31–33]. Similarly, expression of CK5 and TAZ are associated to mesenchymal phenotype [34, 35]. Our results indicated that miR205 repressed the mesenchymal phenotype of SUM159, although re-expression of E-cadherin was not detected. In renal cancer cells and tumors, miR205 binds to the 3’-UTR regions and suppresses expression of several SFKs members [23]. Also in MDA-MB-231 and Hs578t TNBC cell lines, there is an inverse correlation between miR205 and c-Src expression [36]. In SUM159 cells we detected reduced expression of Fyn and Lyn A/B. All these effects were specific, as they were reversed by Anti-miR205 co-expression in SUM159 cells.

The reduced proliferation detected in SUM159 after ectopic expression of miR205 could be correlated to a direct effect on specific signaling pathways. Indeed, activation of Src/Stat3 axis, which is important for Myc and cyclin D1 expression, and cell proliferation [37, 38], was inhibited in SUM159_miR205, cyclin D1, and c-Myc levels were reduced, and p27^Kip1^ expression was augmented. Furthermore, anchorage-independent growth, which in cancer cells correlates with tumorigenic capabilities, was strongly inhibited in SUM159_miR205. Similarly, in A498, BT549, and in MDA-MB-231 cells, miR205 diminishes cyclin D1 and Myc levels, suppresses cell proliferation and colony formation [23, 39].

In addition, accordingly with reduced activation of SFKs (pY418-Src) and Stat3 (pY705-Stat3), miR205 significantly inhibited SUM159 migration and invasion. Consistently, levels of MMP9 in the secretome of SUM159_miR205 cells were also reduced. All these effects were reverted by Anti-miR205 co-expression. In this context, miR205 inhibits expression of several SFKs members, as well as the pSrc/pStat3 axis, cell migration and invasion in A498 cells [23]. Moreover, the suppressive effect of miR205 in ZEB1 expression observed in SUM159_miR205, could be associated with reduced migration and invasion, as it was also detected in MDA-MB-231, MDCK, and esophageal squamous cell carcinoma cells [9, 30, 40].

The main complication of cancer is metastasis and relapse. Within a tumor there is a small subpopulation of cancer-initiating/cancer stem cells responsible for tumorigenesis and cancer maintenance [15, 26, 27]. These cells are usually resistant to conventional chemotherapy, and can remain in a state of latency for long periods of time, making their elimination quite difficult [41]. The breast cancer-stem cells are able to self-renewal forming mammospheres under special culture conditions [42]. Here we showed that miR205 expression in SUM159 inhibited mammospheres formation, as SFE was significantly reduced, indicating that cancer-stem cell renewal was impaired. Consistently, ALDH1 and CD44 cancer-stem markers [43, 44] were also reduced by miR205, as well as TAZ, which is involved in the cancer-stem cells renewal and mammospheres formation [45], and E2A.E12 that maintains the mesenchymal characteristics of this cellular subpopulation. Thus, miR205 by inhibiting cancer-stem cell renewal might affect SUM159 tumorigenic capabilities.

## Conclusions

To summarize, our results strongly suggest that ectopic expression of miR205 in SUM159 cells inhibited cancer stem cell renewal, as well as other parameters associated with initial steps of tumorigenesis, making miR205 a potential powerful therapeutic tool for TNBC treatment.

## Supporting information

S1 Table Detailed information for antibodies used in this work. (PDF)
