# Supplementary material for: miR205 inhibits stem cell renewal in SUM159PT breast cancer cells
Source: PLoS One. 2017 Nov 28;12(11):e0188637. doi: 10.1371/journal.pone.0188637 (PMC5705145; doi:10.1371/journal.pone.0188637)
Supplement: S1 Table — (PDF) [file pone.0188637.s001.pdf]

S1 Table Detailed information for antibodies used in this work.

Name	Type	Supplier	Catalog #	Origin	Dilution Ratio IB*
c-Myc (N-262)	rabbit polyclonal	Santa Cruz Biotechnology, Inc.	Sc-764	Dallas, TX, USA	1:1000
β-Actin	mouse monoclonal	Sigma-Aldrich (Merck)	A5441	San Luis, MI, USA	1:1000
$p27^{Kip1}$ (Clone G173-524)	mouse monoclonal	BD-Biosciences	#554069	Franklin Lakes, NJ, USA	1:1000
c-Src (Src 2)	rabbit polyclonal	Santa Cruz Biotechnology, Inc.	sc-18	Dallas, TX, USA	1:1000
Cyclin D1 (H-295)	rabbit polyclonal	Santa Cruz Biotechnology, Inc.	sc-753	Dallas, TX, USA	1:500
pY418-c-Src	rabbit polyclonal	Invitrogen	#44660G	Camarillo, CA, USA	1:100
ALDH1	mouse monoclonal	BD-Biosciences	#611194	Franklin Lakes, NJ, USA	1:1000
ErB-3 (C-17)	rabbit polyclonal	Santa Cruz Biotechnology, Inc.	sc-285	Dallas, TX, USA	1:1000
Lyn A/B (H-6)	mouse monoclonal	Santa Cruz Biotechnology, Inc.	sc-7274	Dallas, TX, USA	1:1000
Fyn (FYN3)	rabbit polyclonal	Santa Cruz Biotechnology, Inc.	sc-16	Dallas, TX, USA	1:1000
VEGF-A (A-20)	rabbit polyclonal	Santa Cruz Biotechnology, Inc.	sc-53462	Dallas, TX, USA	1:1000
E2A.E12	rabbit polyclonal	Santa Cruz Biotechnology, Inc.	sc-133075	Dallas, TX, U.S.A	1:500
Zeb-1 (E-20)	goat-polyclonal	Santa Cruz Biotechnology, Inc.	sc--10572	Dallas, TX, USA	1:1000

Stat3	mouse monoclonal	BD-Biosciences	S21320	Franklin Lakes, NJ, USA	1:2000
pY705-Stat3	rabbit polyclonal	Cell Signaling Technology, Inc.	#9131	Danvers, MA, USA	1:1000
Twist 1/2	rabbit polyclonal	Gene Tex, Inc.	GTX127310	Irvine, CA, USA	1:1000
CK5	rabbit polyclonal	ABCAM	ab52635	Cambridge, UK	1:5000
GAPDH (clone 6C5)	mouse monoclonal	Millipore (Merk)	CB1001	Billerica, MA, USA	1:4000
PARP (Clone C-2-10)	mouse monoclonal	Biomol GmbH	SA-249	Hamburg, Germany	1:1000
TAZ (WWTR1)	rabbit polyclonal	Santa Cruz Biotechnology, Inc.	HPA007415	Danvers, MA, USA	1:250
Snail 1	mouse monoclonal	Cell Signaling Technology, Inc.	LF062	Danvers, MA, USA	1.100
CD44 (Clone HP 2/9)	mouse monoclonal	Gift from Dr, Sanchez-Madrid		Madrid, Spain	1:1000
MMP9	rabbit polyclonal	Millipore (Merk)	AB19016	Billerica, MA, USA	1:1000

***, Immunoblotting (IB)**

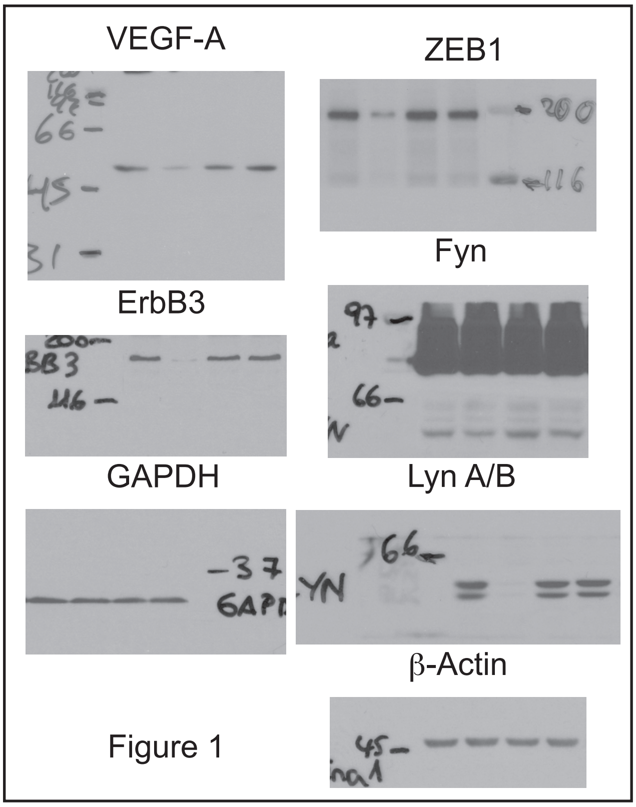

Figure 1

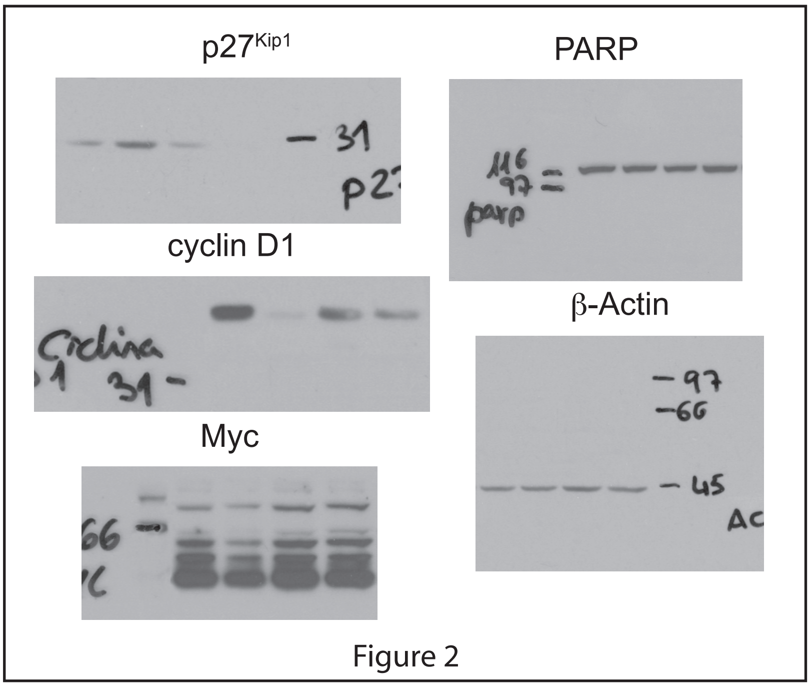

Figure 2

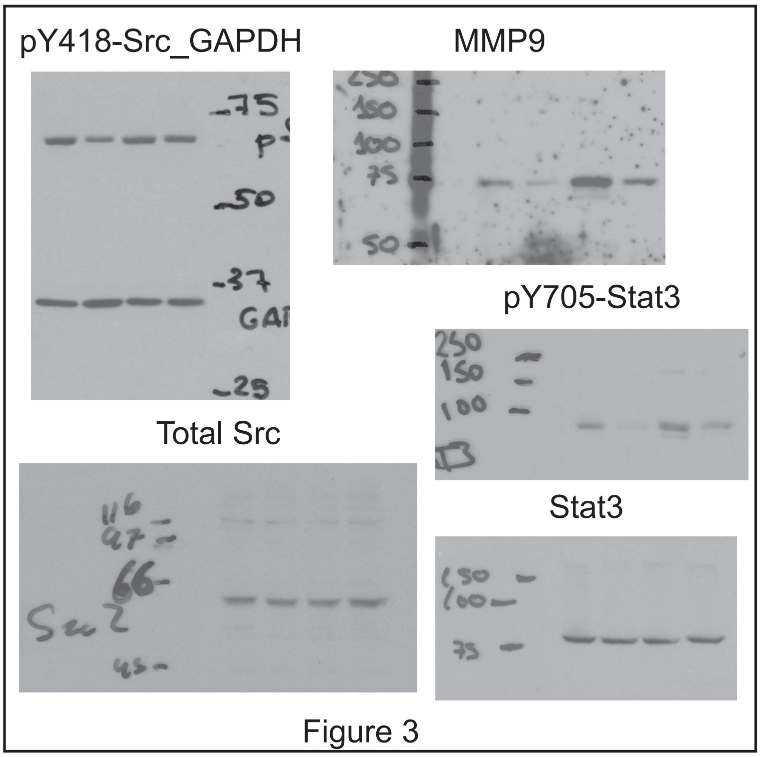

Figure 3

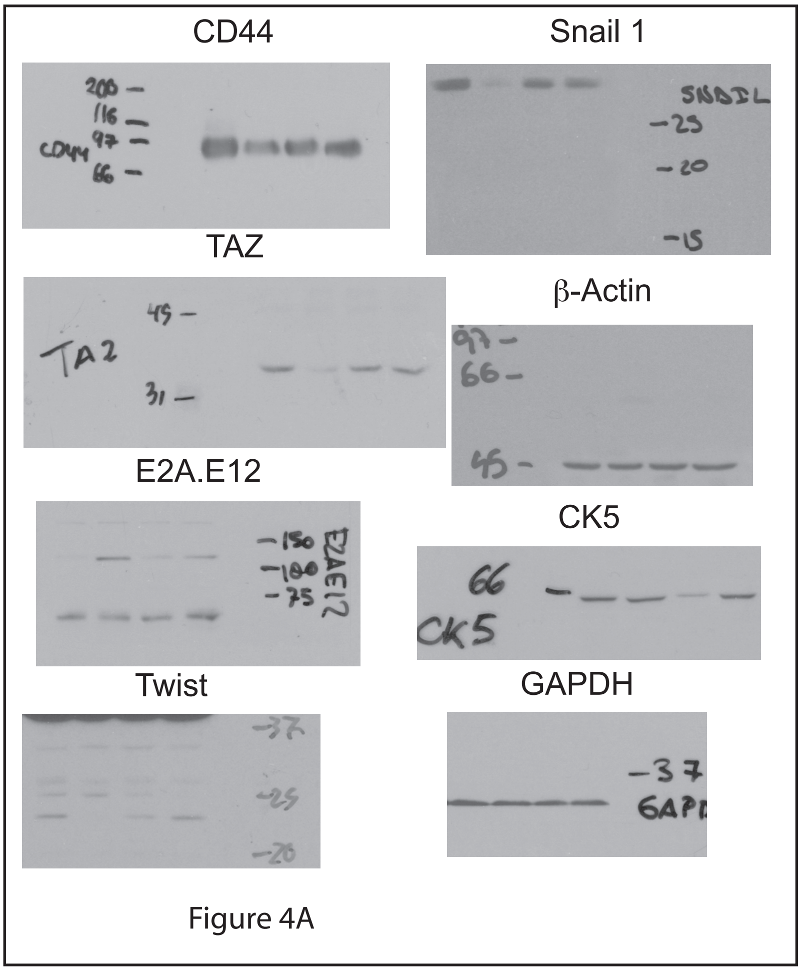

Figure 4A

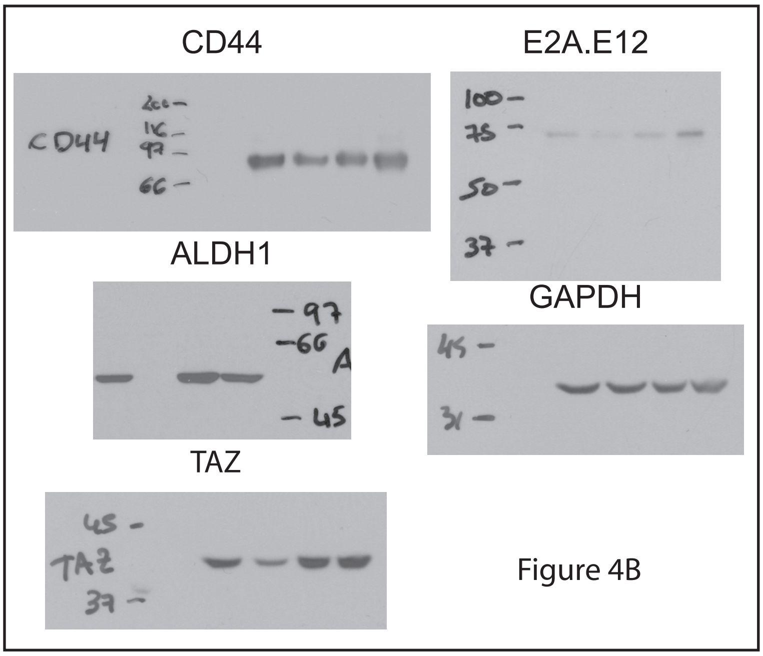

Figure 4B
